# Massive splenomegaly requiring differential diagnosis of hematologic malignancies

**DOI:** 10.1002/ccr3.5512

**Published:** 2022-03-10

**Authors:** Masaki Tago, Motoshi Fujiwara, Tomotaro Nakashima, Seijiro Makio, Yuka Hirakawa, Midori Tokushima, Shun Yamashita, Yoshinori Tokushima, Hidetoshi Aihara, Shu‐ichi Yamashita

**Affiliations:** ^1^ Department of General Medicine Saga University Hospital Saga Japan

**Keywords:** massive splenomegaly, splenic marginal zone lymphoma

## Abstract

An 85‐year‐old woman presented with pain and a palpable mass in her left flank. Abdominal computed tomography revealed massive splenomegaly and para‐aortic lymphadenopathies. Bone marrow biopsy showed CD79a, CD20, and bcl‐2‐positive atypical lymphocytes, which led to the diagnosis of splenic marginal zone lymphoma.

## Graphical abstract

The causes of massive splenomegaly are hematologic diseases, malignancies, infection, and infiltrative conditions. When seeing massive splenomegaly, hematologic diseases, especially malignant diseases, should be ruled out first.

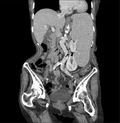

## 1 CASE

**FIGURE 1:**
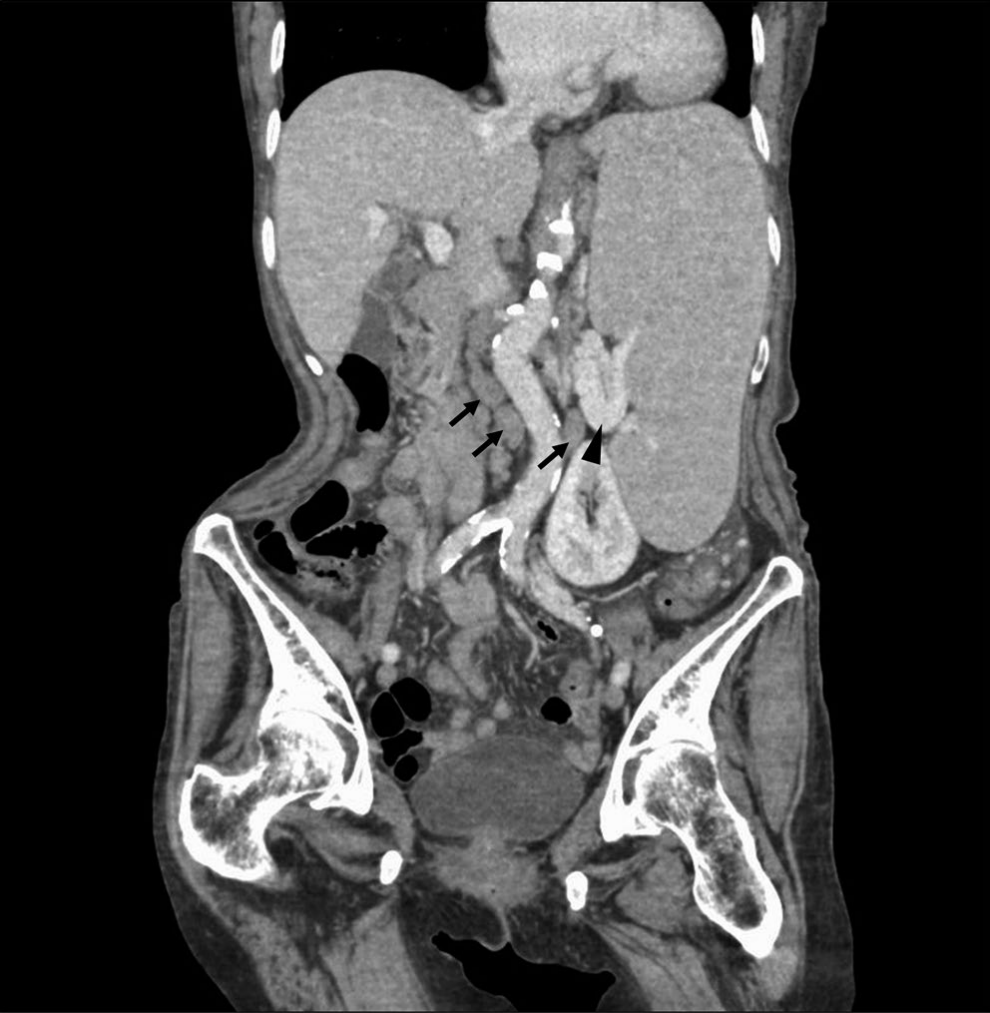
Abdominal contrast‐enhanced computed tomography findings. Abdominal contrast‐enhanced computed tomography images showing massive splenomegaly with a dilated splenic vein (arrowhead) and enlarged para‐aortic lymph nodes (arrows)

An 85‐year‐old woman with good appetite, no fever or weight loss visited our hospital because of left flank pain. Physical examination revealed a palpable mass with tenderness in her left flank, no palpable lymph nodes in any area of the body, and no other abnormal findings. Blood examination revealed: white blood cell count: 6.1 × 10^9^/L, hemoglobin: 106 g/L, platelets: 9.7 × 10^9^/L, soluble interleukin‐2 receptor 4,006 U/mL, and no abnormalities in liver function, including lactate dehydrogenase. Abdominal contrast‐enhanced computed tomography revealed massive splenomegaly of >20 cm without mass lesion, and para‐aortic lymphadenopathies (Figure 1, Videos S1 and S2). Bone marrow biopsy revealed proliferation of atypical lymphocytes, and immunostaining was positive for Cluster Designation (CD)79a, CD20, and bcl‐2, which led to a diagnosis of splenic marginal zone lymphoma (SMZL).

SMZL is a rare subtype of non‐Hodgkin lymphoma showing splenomegaly and lymphocytosis.^1^ The accurate diagnosis of SMZL is delayed because it does not show remarkable clinical symptoms and aggressive clinical courses.^1^ The causes of massive splenomegaly are leukemias, non‐Hodgkin lymphoma, myelofibrosis, metastatic cancer, primary splenic tumors, infection, autoimmune hemolytic anemia, β‐thalassemia major, megaloblastic anemia, hereditary spherocytosis, and infiltrative conditions.^2^ When seeing massive splenomegaly, hematologic malignant diseases should be ruled out first.

## CONFLICT OF INTEREST

The authors have declared no conflict of interest.

## AUTHOR CONTRIBUTIONS

Tago M was involved in the conception of the study, and in the literature search and drafting the manuscript. Fujiwara M, Tokushima M, Yamashita S, Tokushima Y, and Aihara H were involved in the conception of the study and drafting the manuscript. Nakashima T, Makio S, and Hirakawa Y were involved in the literature search and clinical care of the patient. Yamashita SI was involved in the conception of the study and revising the manuscript.

## ETHICAL APPROVAL

This manuscript conforms to the provisions of the Declaration of Helsinki in 1995 (as revised in Brazil 2013).

## CONSENT

Written informed consent to publish this report was obtained from the patient in accordance with the journal's patient consent policy.

## Supporting information

Video S1

Video S2

Supplementary Material

## Data Availability

The data that support the findings of this study are available from the corresponding author upon reasonable request.
