# Supplementary material for: Massive splenomegaly requiring differential diagnosis of hematologic malignancies
Source: Clin Case Rep. 2022 Mar 10;10(3):e05512. doi: 10.1002/ccr3.5512 (PMC8908078; doi:10.1002/ccr3.5512)
Supplement: Supplementary file 3 — Supplementary Material [file CCR3-10-e05512-s002.docx]

**Supporting information**

**Video of computed tomography scroll-through; Video S1: axial view and S2: coronal view.**
